# Role of Bioactive Peptide Sequences in the Potential Impact of Dairy Protein Intake on Metabolic Health

**DOI:** 10.3390/ijms21228881

**Published:** 2020-11-23

**Authors:** Giovanni Tulipano

**Affiliations:** Unit of Pharmacology, Department of Molecular and Translational Medicine, University of Brescia, 25123 Brescia, Italy; giovanni.tulipano@unibs.it; Tel.: +39-03-0371-7510

**Keywords:** bioactive peptides, food peptides, identification and characterization, whey, enteroendocrine hormones, food intake, appetite and satiety, glucose homeostasis, DPP-IV activity

## Abstract

For years, there has been an increasing move towards elucidating the complexities of how food can interplay with the signalling networks underlying energy homeostasis and glycaemic control. Dairy foods can be regarded as the greatest source of proteins and peptides with various health benefits and are a well-recognized source of bioactive compounds. A number of dairy protein-derived peptide sequences with the ability to modulate functions related to the control of food intake, body weight gain and glucose homeostasis have been isolated and characterized. Their being active in vivo may be questionable mainly due to expected low bioavailability after ingestion, and hence their real contribution to the metabolic impact of dairy protein intake needs to be discussed. Some reports suggest that the differential effects of dairy proteins—in particular whey proteins—on mechanisms underlying energy balance and glucose-homeostasis may be attributed to their unique amino acid composition and hence the release of free amino acid mixtures enriched in essential amino acids (i.e., branched-chain-amino acids) upon digestion. Actually, the research reports reviewed in this article suggest that, among a number of dairy protein-derived peptides isolated and characterized as bioactive compounds in vitro, some peptides can be active in vivo post-oral administration through a local action in the gut, or, alternatively, a systemic action on specific molecular targets after entering the systemic circulation. Moreover, these studies highlight the importance of the enteroendocrine system in the cross talk between food proteins and the neuroendocrine network regulating energy balance.

## 1. Introduction

For years, there has been an increasing move towards harnessing the health-promoting benefits of traditional foods and novel foods while providing scientific evidence to substantiate their claims. In particular, the potential for bioactive compounds from foods to modulate hunger, satiety, body weight gain and plasma glucose levels is being investigated [1,2,3].

Food proteins are a well-recognized source of bioactive compounds, and dairy foods can be regarded as the greatest source of proteins and peptides with various purported health benefits, their effects ranging from benefitting the digestive system, cardiovascular circulation, the immune system, the central nervous system and the neuroendocrine control of energy homeostasis [4,5]. 

Focusing on the last target, a number of dairy protein-derived peptides with the ability to modulate food intake, body weight gain and glucose homeostasis have been isolated and characterized. Their being active in vivo may be questionable and needs to be discussed [6,7,8]. 

The ingestion of dietary proteins exerts a strong suppressive effect on hunger and energy intake [5,7,9]. In fact, proteins are considered more satiating compared to an isoenergetic intake of other macronutrients, i.e., carbohydrates. Actually, the link between protein consumption, food intake and control of body weight is not without controversy. In detail, it has been not clearly established as to whether the satiating effect of dietary proteins depends solely on the release of free amino acids after their extensive hydrolysis by pepsin in the stomach and by pancreatic and brush border proteases in the small intestine [10], while it may also depend on the release of functional peptides. To this end, after the hydrolysis by pancreatic enzymes, about 70% of ingested nitrogen is found in the form of peptides, of which most of them are between two and fourteen amino acids long, according to the analysis of jejunal samples [11,12]. 

**Figure 1:**
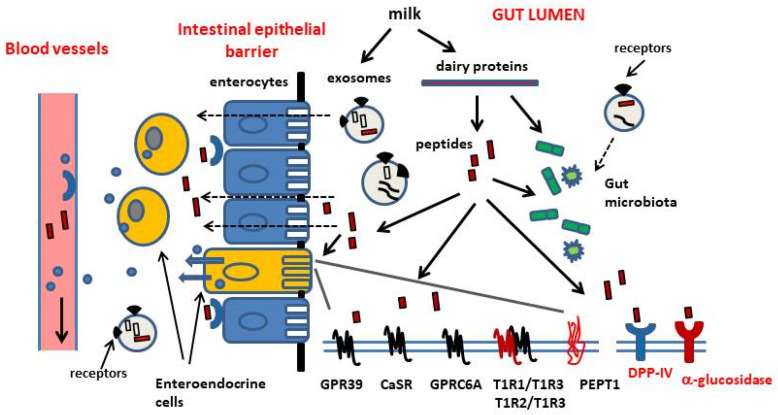
Schematic overview of some bioactive components that are released in the gastrointestinal tract upon intake and digestion of milk and dairy products, and their cellular and molecular targets at the level of the gut lumen and the gut wall. Digestion products of dairy proteins include free amino acids (not shown) and peptides. Some peptides act as signaling molecules and regulate the activity of enteroendocrine cells exposed to the gut lumen through binding to G-protein coupled receptors (GPR39, CaSR, GPRC6A, T1R1/T1R3 and T1R2/T1R3 heterodimers) and peptide transporters (PEPT1), at the level of their apical membrane. Some peptides act as inhibitors of dipeptidylpeptidase-IV (CD26/DPP-IV) activity and alpha-glucosidase activity, at the level of the brush border of enterocytes. On the other hand, the up-regulation of DPP-IV expression has been associated with the ingestion of the genetic A1-variant of beta-casein. Dairy proteins and their peptidic fragments (i.e., glycomacropeptide, GMP) are also believed to influence the composition of the gut microbiota. Some bioactive peptides can be transported across the intestinal epithelial barrier and into circulation. At the level of the basolateral pole of the enteroendocrine cells, the DPP-IV inhibitory peptides can reduce the degradation of glucagon-like peptide-1 (GLP-1) and glucose-dependent insulinotropic peptide (GIP), thus increasing their release into circulation. Bioactive peptides acting on endocrine cells not exposed to the gut lumen (ghrelin-secreting cells and somatostatin-secreting cells) have been also described. Finally, exosomes are a subpopulation of so called extracellular vesicles (EVs, submicron-sized lipid envelops). EVs are believed to mediate cellular signalling by carrying nucleic acids, proteins, lipids and cellular metabolites between cells and organs. Exosomes contain macromolecules like peptides, mRNA and microRNAs and can interact with human cells. Exosomes have been isolated from bovine milk and also from whey protein hydrolysates. Dietary exosomes may be considered a novel factor *per se* in the functional interaction between foods and biological systems. Bovine milk exosomes significantly alter the gut microbiome in non-bovine species. Although there is no evidence, yet of food-derived exosome absorption into circulation in vivo, it would be interesting to investigate a possible role of exosomes as a delivery system protecting bioactive peptides from degradation and facilitating their passage across the intestinal barrier and their delivery to tissue targets.

Various mechanisms mediate the appetite-suppressant and weight-reducing effects of dietary proteins, as well as their role in plasma glucose homeostasis. These mechanisms include actions of digestion products on the central nervous system (CNS), peripheral nervous system and endocrine system, on the condition that they can be transported across the intestinal barrier into circulation. In this way, the ingestion of dietary proteins can affect hormone secretion and energy expenditure via increased thermogenesis, the homeostatic regulation of food intake at the hypothalamic level and cell metabolism at the skeletal muscle and liver levels [1,7,13,14,15,16,17,18]. The bioavailability of bioactive peptides derived from protein hydrolysis has been in dispute for a long time and the increase of free amino acids levels in blood is believed to play a major role in this context [10,19,20,21]. Actually, it is well known that the mucosa of the gastrointestinal tract (GI) is strongly involved in the management of energy balance via the actions of dispersed enteroendocrine cells that release signals, mainly peptidic hormones and serotonin which regulate the rate of gastric emptying, small bowel motility, pancreatic enzyme and pancreatic hormone secretion. Moreover, these signals are the main connection between the ingestion and GI digestion of foods, and the neuroendocrine network regulating energy intake, glucose homeostasis and cellular bioenergetics [21,22,23,24]. Most enteroendocrine cells dispersed in the mucosa of the GI tract can directly sense nutrients at their apical pole facing the gut lumen, and can be induced to release hormones from their basal pole into circulation. Receptors belonging to the class of G protein-coupled receptors (GPCRs) have been implicated in protein/amino acid sensing in enteroendocrine cells [12,25]. Actually, intestinal transporters may also be involved in this function [26]. These enteroendocrine cells can be an easy target of functional peptides released after enzymatic digestion of dietary proteins in the gut lumen (Figure 1). Some enteroendocrine cell subtypes (ghrelin-releasing cells, somatostatin-releasing cells and enterochromaffin cells) do not make contact with the gut lumen [12]. 

All dietary proteins most likely exert a direct stimulatory effect on satiety hormone release upon digestion in the gut and, to our knowledge, this effect has not been related to any specific secondary or tertiary structure features. Indeed, also free amino acids can exert similar effects on enteroendocrine cells and pancreatic β-cells [21,22,27,28]. Nevertheless, in recent years, it has been shown that some differences may exist between distinct dietary proteins in their secretory activity in vitro and their satiating or appetite-suppressant effects in vivo. If these actions were related solely to the release of free amino acids upon extensive digestion in the gut lumen, then the differential activity of distinct proteins should be attributed to their unique amino acid composition, in detail the relative content of specific amino acids. Actually, some in vitro studies showed that intact proteins and partially hydrolyzed proteins were more effective at inducing enteric hormone secretion than the products of their complete hydrolysis, and some selected synthetic peptides were more effective than the corresponding free amino acids [29,30]. In conclusion, distinct features in the structure of a given dietary protein may positively affect its ability of interacting with surface receptors on the enteroendocrine cell and inducing the release of a given hormone. These features would not be resistant to extensive digestion of proteins. On the other hand, they may be retained by small peptides released during hydrolysis of proteins. In summary, not only the relative content of specific amino acids, but also the spatial proximity between amino acids residues, may be relevant to this functional activity of dietary proteins. Ascertaining the actual role played by free amino acids versus bioactive peptides in the interaction of dietary proteins with the enteroendocrine system may be useful to prepare nutraceuticals to be included in a dietetic regimen aimed at controlling body weight gain or to help glucose homeostasis.

### 1.1. Nutrient-Sensing Receptors

So-called nutrient-sensing receptors are specialized receptors expressed by cells within the GI wall, which recognize the products of food digestion (Figure 1). Among the G-protein coupled receptors (GPCRs) mediating the stimulatory effects of amino acids and peptides on GI hormone release, there are taste receptors of the T1R family, GPRC6A, Calcium-Sensing Receptor (CaSR) and GPR93 (also known as GPR92 or LPAR5) [12,25]. These receptors mediate nutrient sensing and transmit signals inside different subtypes of enteroendocrine cells within the gastrointestinal mucosa, to regulate their activity. The T1R1/T1R3 and T1R2/T1R3 heterodimers and the calcium sensing receptor (CaSR) and GPRC6A receptor, both thought to be expressed as homodimers, can detect amino acids in the extracellular milieu and show partial selectivity for ligands [25]. Distinct amino acids can bind to the same nutrient-sensing receptor but they activate differential transduction pathways. The activation of T1R1/T1R3 receptor by l-leucine, l-phenylalanine, l-glutamate was shown to induce the release of cholecystokinin (CCK) in enteroendocrine cells (STC-1 cell line) [25,28]. This receptor is also involved in the regulation of GLP-1 and peptide YY secretion. CaSR mainly senses aromatic amino acids [26,31] and was shown to mediate the l-phenylalanine- induced CCK secretion in STC-1 cells [32]. In addition to free amino acids, some basic amino acid-rich peptides and γ-glutamyl peptides have been identified as CaSR agonists [12]. Basic amino acids are the most potent agonists of mouse GPRC6A, but this receptor is also activated by l-alanine and l-serine [25,33,34]. Moreover, GPRC6A is believed to mediate some effects of the bone-derived peptide osteocalcin on hormone secretion from pancreatic β-cells and from STC-1 cells [35]. Finally, GPR39 is highly expressed in enteroendocrine cells in the stomach and the small intestine and is involved in the control of gastrin- and CCK-secretion. Its cellular levels are increased in response to a high-protein diet intake. It is believed to be activated by peptides to a higher extent than by free amino acids [12,36].

Nutrient-sensing receptors have large ligand-binding segments that can accommodate a variety of molecules acting as agonists, allosteric activators or inhibitors. In addition to amino acids, GPRC6A and CaSR are activated by calcium and other inorganic cations [37,38,39]. In fact, calcium is an essential co-factor for CaSR activation by free amino acids and peptidic ligands, which are ineffective in the absence of calcium. Receptors of the T1R family were initially identified as gustatory taste receptors. The TR1R2/T1R3 heterodimer is activated by a broad range of sweet compounds. The T1R1/T1R3 complex is the umami receptor [25].

In addition to receptors belonging to the GPCR class, nutrient-sensing is also dependent on amino acid- and peptide-transporters. PEPT1 is a di- and tripeptide intestinal transporter, also expressed in the STC-1 cell line. It has been associated with the control of CCK and GLP-1 secretion [26].

### 1.2. Gastroenteric Hormones

The main peptidic hormones released into circulation by the enteroendocrine cells in the gut in response to an oral nutrient load include peptide CCK, Tyrosine-Tyrosine (PYY), oxyntomodulin, glucagon-like-peptide-1 (GLP-1) and glucose dependent insulinotropic peptide (GIP) [22,23,27]. On the other hand, ghrelin is an orexigenic peptide released in response to fasting [40]. Enteroendocrine cells do not exceed 1% of all intestinal mucosa epithelial cells. Distinct subtypes of enteroendocrine cells differ in the hormones they can release and in their localization along the GI tract. A more detailed list and description of the enteroendocrine cell subtypes in the GI tract and the related GI hormones, can be found in the review paper by Santos-Hernandez et al. [12]. 

**Figure 2:**
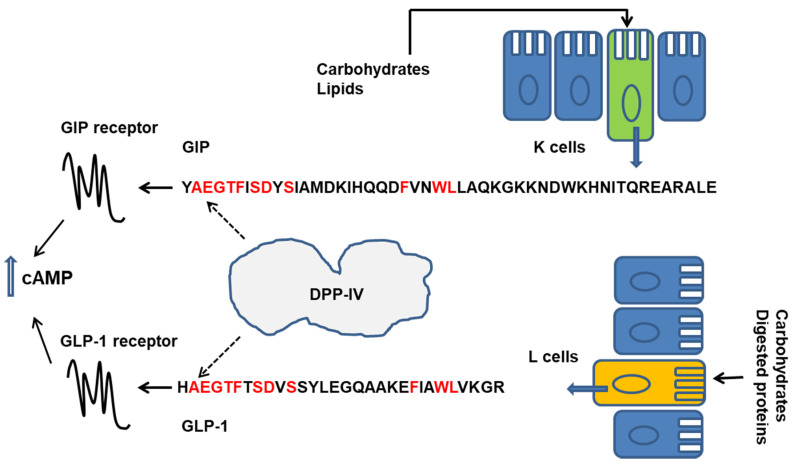
Glucagon-like peptide-1 (GLP-1) and glucose-dependent insulinotropic peptide (GIP), so called incretin hormones, are secreted by distinct enteroendocrine cells, K cells in the proximal bowel and l cells in the ileum and colon, respectively, in response to food digestion products. The aminopeptidase dipeptidylpeptidase-IV (DPP-IV) rapidly deactivates GLP-1 and GIP by removing their *N*-terminal dipeptides. GIP and GLP-1 share some amino acids in their sequences. They activates two distinct G-protein coupled receptors (GPCRs), which both activate adenylyl cyclase (AC), thus increasing cyclic adenosinmonophosphate (cAMP) levels in target cells. GIP and GLP-1 are well-known glucose-dependent insulinotropic peptides. They also promote satiety and reduce appetite and contribute to regulate gastric emptying and acid secretion. The enzymatic digestion of dietary proteins cause the release of free amino acids and peptides that can stimulate GLP-1 secretion by activating nutrient-sensing receptors expressed by l cells [12]. Peptidic inhibitors of the α-glucosidase activity may also have an impact on GLP-1 secretion after a meal. Indeed, the enzyme inhibition delays the monosaccharide absorption, with the outcome of an increase in free sugar levels in the lower intestine, which in turn may enhance the release of GLP-1 from l cells Finally, evidence has been accumulated that the enzymatic digestion of dietary proteins can produce peptidic DPP-IV inhibitors that have the potential to slow-down incretin cleavage, thus enhancing the biological activities of both GLP-1 and GIP. Moreover, there is increasing interest in a possible effects of food derived compounds on DPP-IV expression in the gut [53].

CCK is a gastrointestinal peptide secreted by enteroendocrine cells in the proximal small intestine in response to luminal proteins. CCK cooperates with the signalling networks regulating gall bladder contraction, the secretion of pancreatic enzymes, the rate of gastric motility and emptying, and appetite and food intake [9,41,42]. The release of CCK is induced by the products of protein hydrolysis in the gut, including small peptidic fragments and free amino acids, and by lipids [25,26]. GLP-1 and GIP, so-called incretin hormones, enhance insulin secretion and suppress glucagon secretion after a meal, thus contributing to plasma glucose homeostasis. They also slow gastric emptying and reduce food intake [43]. GIP and GLP-1 are secreted by distinct enteroendocrine cells, K cells in the proximal bowel and l cells in the ileum and colon, respectively. l cells also release peptide YY and are regarded as to be more exposed to slowly digested nutrients and products of bacterial fermentation [27]. GLP-1 and GIP are rapidly inactivated by the aminopeptidase dipeptidylpeptidase-IV (DPP-IV), which removes their *N*-terminus. DPP-IV exists as a cell-surface enzyme in numerous cell types (i.e., endothelial cells) and in the brush border of intestinal mucosa, and also exists as a soluble circulating form [44]. A rapid degradation of incretins is expected after their release, even before they leave the GI tract [45,46,47,48]. In the last decade, evidence has been accumulated that the enzymatic digestion of dietary proteins can produce peptidic DPP-IV inhibitors that have the potential to slow-down incretin cleavage, thus enhancing their biological activities [49,50,51,52]. Moreover, there is increasing interest in a possible effects of food derived compounds on DPP-IV expression in the gut [53] (Figure 2).

Ghrelin is a peptidic hormone that was first isolated from the fundus of the stomach. Actually, ghrelinergic cells have been found in different tissues, which include the enteric mucosa and pancreatic islets. Among gastroenteric hormones, ghrelin is an orexigenic peptide, secreted in response to starvation, responsible of the feeling of hunger and affecting feeding behavior [54,55]. Actually, ghrelin is not unique in this function. More recently, a second orexigenic gut hormone has been identified. Insulin-like peptide 5 (Insl5) is a member of the relaxin family of peptides isolated from colonic tissue and neuroendocrine tumors. As various neuroendocrine peptides, Insl5 is likely to exert multiple actions in different tissues. In the context of the enteroendocrine system, Insl5 is released by a subset of colonic l cells in response to fasting or prolonged calorie restriction and stimulates food intake in rodents. Hence, Insl5 joins the gut hormone system and contributes to link food ingestion to nutrient transit across the GI tract [56].

## 2. How Dairy Proteins Can Modulate Food Intake and Glucose Homeostasis

### 2.1. Role of Free Amino Acid as Metabolic Signals

Diets lower in carbohydrate and higher in protein content have shown promising results on short-term weight loss and glycemic control [7,57]. Actually, concerns have been raised about potential adverse effects of long-term high protein intake [58,59,60]. Major nutrition recommendations for patients affected by a metabolic disease like type 2 diabetes mellitus, have been reported elsewhere. Individualized medical nutrition therapy and mixed meals have been indicated [61]. 

There has long been interest in a possible differential impact of distinct dietary protein sources on food intake regulation and glucose homeostasis. To this end, epidemiological studies demonstrate that the consumption of dairy foods, i.e., low fat dairy, milk and yogurt can help to maintain a healthy body weight and metabolic health [62]. Dairy proteins, especially the whey protein fraction, seem to play a main role [63,64,65,66,67,68]. 

The characterization of specific dietary proteins with a high impact on metabolic health may be useful to produce supplements or functional foods to be included in a mixed dietetic regimen of a body weight reduction program or to achieve a better glycemic control after meal [3,4,8]. 

Dietary proteins are basically a source of free amino acids and may differ in their amino acid composition, that means in the relative content of a given amino acid. High-quality proteins, or complete proteins, contain substantial amounts of all essential amino acids. Apart from being building blocks of structural and functional proteins, the amino acids play a direct role in the control of metabolism at different levels.

The release of amino acids by food protein digestion enhance the secretion of hormones from the enteroendocrine cells in the GI tract, which tended to reduce further food consumption and cooperate to the control of postprandial glycemia. The role played by nutrient-sensing receptors exposed on the apical cell surface in the gut lumen, has already been mentioned [22,25,28,32,33,34]. Moreover, circulating amino acids regulate the secretion of hormones (insulin, glucagon, growth hormone) involved in the control of energy balance. Some amino acids are insulinotropic agents, the most potent secretagogue being l-arginine, l-lysine, l-leucine and l-phenylalanine. Actually, despite the fact that circulating amino acids can be regarded as insulin secretagogues, the impact of an increase in plasma free amino acids on the regulation of plasma glucose levels is still controversial because amino acids have multiple roles in cells. More precisely, distinct amino acids may differ in their effects on glucose metabolism and tissue response to insulin, and branched-chain amino acids (l-leucine, l-isoleucine, l-valine) have been extensively investigated for their metabolic impact [19,61,69].

Free amino acids have the potential to reduce the glucose utilization due to the preferential oxidation of amino acids versus glucose, thus reducing the glucose tolerance [69]. The plasma levels of branched-chain amino acids (BCCAs) have been found higher in obese subjects compared to lean subjects and obesity is a risk factor for type 2 diabetes development [70,71,72]. Furthermore, the l-leucine deprivation increased insulin sensitivity in in vitro hepatocytes [72] and the acute exposure to some amino acids (l-leucine, l-methionine, l-threonine and l-lysine) reduced the insulin-induced glucose transport in skeletal muscle cells by interfering with the phosphoinositide-3-kinase (PI3K)-mediated signalling downstream of insulin receptor [73,74]. On the other hand, higher BCAAs intake has been associated with a lower prevalence of being obese in middle-aged subjects [75] and a long-term supplementation with an amino acid formula improved glycemic control in elderly subjects with type 2 diabetes [76]. Preclinical studies showed an improvement of glucose tolerance in response to l-leucine in synergy with insulin action in mice [77], and a BCAA mixture enhanced the glucose transport even in the absence of insulin in different in vitro experimental models of skeletal muscle cells [78,79]. Finally, a specific BCAA-containing mixture increased the mitochondrial biogenesis and preserved the skeletal muscle and cardiac tissue from oxidative damage in mice [80]. In this context, a more recent in vitro study showed that some products of simulated GI digestion of whey proteins, could transit across a Caco-2 cell monolayer and inhibited free radicals formation in muscle cells. These products consisted of free amino acids, with notable levels of BCAAs and sulphur-containing amino acids derived from β-lactoglobulin (β-LG), and also some peptides that were transported across the intestinal epithelial cell barrier and reduced oxidative stress in muscle cells [81]. In summary, both positive and negative effects of amino acid supplements on glucose tolerance, have emerged from preclinical and clinical studies. The composition of an amino acid supplement or the relative content of distinct amino acids in a given dietary protein when used as functional food, in conjunction with the duration of treatment, may determine the final impact on metabolic health. 

A final consideration may concern possible risks related to high protein- or free amino acid mixture intake. An excess of intracellular free amino acids has been shown to lead to overactivation of the mammalian-target-of-rapamycin (mTOR), signalling cascade in skeletal muscle cells. This action, in conjunction with the insulinotropic activity of some amino acids, is believed to mediate the anabolic effects and the increased mitochondrial biogenesis in skeletal muscle tissue, associated with this kind of dietary intervention [80]. Actually, according to a consensus related to the discovery and development of safe interventions to increase a healthy lifespan published by experts in the biology and genetics of aging [60], the possible strategies believed to be most promising include the pharmacological inhibition of the mTOR-S6 protein kinase signalling pathway and strategies aimed at improving the control of plasma glucose levels through insulin-sensitizing agents rather than insulin secretagogues, as mechanisms of protection from cancer and age-related chronic diseases. In summary, if not tissue-selective, the overactivation of the mTOR pathway may represent a drawback associated with a high protein diet or an amino acid supplementation.

Focusing on the appetite-suppressant effect related to food protein intake, it is worthy reviewing a recent research report on the short-term effects of whey protein administration in obese subjects [10]. Whey proteins are high nutritional quality proteins and are regarded as the most effective at reducing appetite and food intake among different sources of proteins. Furthermore, a number of preclinical and clinical studies suggest that whey protein consumption has the potential to improve postprandial glycemic control and to reduce the incidence of insulin resistance associated with being overweight [49,63,64,65,66,67,82,83,84,85,86]. 

There is substantial agreement that the effects of whey protein intake on appetite and food intake are mediated by stimulatory effects on the release of satiety hormones from enteroendocrine cells in the GI tract (in detail, CCK, GLP-1, peptide YY) and insulin from pancreatic β-cells [7,8,10,16,17,30]. It is still in dispute whether the release of functional peptides interacting with the enteroendocrine system or, alternatively, the rise in the plasma concentrations of specific amino-acids upon whey protein digestion, plays a major role in the above-mentioned activity. The article published by Rigamonti and coworkers [10] showed that the appetite-suppressant and GLP-1-stimulating effects of whey protein intake in obese subjects were associated with increased circulating levels of eight specific amino acids (l-isoleucine, l-leucine, l-lysine, l-methionine, l-phenylalanine, l-proline, l-tyrosine and l-valine), thus remarking the relevance of the unique amino acid composition of whey proteins, which include the high content of BCAAs. The plasma levels of CCK were not reported on. We may remark that the eight amino acids include activators of distinct nutrient-sensing receptors and insulin secretagogues (see previous section). 

Elovaris and co-workers studied the plasma free amino acid responses to whey proteins in lean healthy men and showed that the increase in appetite- and gluco-regulatory hormones levels (CCK, GLP-1, insulin) strongly correlated with BCAAs as well as l-lysine, l-methionine, l-aspartic acid, l-tryptophan and l-tyrosine. On the other hand, only a weak inverse correlation was observed between energy intake and free amino acid levels suggesting that multiple factors mediate the effects of protein intake on appetite and hunger in lean subjects [87]. 

Apart from the amino acid composition, the unique fate of whey proteins in the GI tract compared with other proteins (i.e., caseins) may contribute to their effects. Whey proteins are so-called “fast” proteins, based on the rate of amino acid absorption and the effects on endogenous protein synthesis. After being ingested, whey proteins reach the jejunum rapidly as intact polypeptides to be digested slowly in the small intestine, compared with other proteins. On the other hand, caseins are prone to clot in the stomach and their release to the intestine is delayed. Despite longer digestion in the intestine, the degradation of whey proteins and the amino acid absorption is still faster than for proteins like caseins (“slow” proteins). The outcome is a faster and higher, but short-lasting rise of plasma amino acids, including BCAAs [13]. Actually, the peculiar fate of whey proteins in the GI, with the lack of an extensive peptic hydrolysis, may also facilitate the interaction of intact or partially digested polypeptides with the intestinal mucosa, and the release of functional peptidic fragments able to interact with enteroendocrine cells over a great length of intestine before being fully degraded.

In conclusion, the above-mentioned reports suggest that the differential effects of distinct dietary proteins on appetite and hunger may be attributed to the unique amino acid composition of a specific protein. In agreement with this conclusion, the effects of whey protein intake on measures of satiety in normal weight women did not significantly differ from those of an amino acid mixture mimicking the composition of whey proteins [20]. However, these studies cannot rule out a functional interaction between specific peptides derived from enzymatic hydrolysis of whey proteins and the systems underlying the control of energy intake, which include the enteroendocrine system, in addition to the effects related to the release and absorption of free amino acids. Indeed, other studies evidenced some differences between the effects of whey protein-based supplements compared to free amino acid-based supplements. In healthy humans, the GIP response to gluco-equivalent drinks was found to be significantly enhanced by the addition of whey proteins, not so by the addition of an amino acid mixture enriched in BCAAs [88]. Moreover, in a study comparing the effects on appetite and energy intake of two distinct dietary supplements matched for the essential amino acid (EAA) content and administered to aged women before breakfast, the whey protein isolate (WPI) pre-load was able to decrease ad libitum energy intake and to increase peptide YY secretion to a higher extent compared to the EAA-based gel pre-load. Furthermore, accounting for the energy content of the two supplements, the whole energy intake in women that were given the EAA-gel was higher compared to both the control group and the WPI group [89]. 

### 2.2. Evidence Supporting a Role of Bioactive Peptides

Bioactive peptides are defined as specific protein fragments (approximately, from two- up to twenty amino acid long) that exert a positive impact on body functions and ultimately have a beneficial effect on human health [4,90,91] Their activity is based on their unique amino acid composition and sequence. Dietary proteins provide a rich source of bioactive peptides. Such peptides are encrypted within the amino acid sequence of a given protein as inactive precursors and can be released in three ways: (a) through hydrolysis by digestive enzymes in the GI tract or during simulated GI digestion in vitro, (b) through hydrolysis by microbial fermentation and (c) through the action of proteolytic enzymes isolated from microorganisms or plants. Bioactive peptides, especially short peptides, can also be synthetized chemically. Chemical synthesis may be a method to prepare large amounts of a specific peptide to be administered in vivo, provided that an effective delivery system is available [2,3,4,8]. Synthetic peptides are also useful to characterize the functional properties associated with a specific amino acid sequence in vitro.

Bioinformatics enables in silico studies aimed at predicting the yield of bioactive peptides from a given dietary protein sequence. Bioinformatics tools include databanks of whole protein sequences, databanks of well-characterized bioactive peptide sequences, software enabling the detection of biologically active fragments in a protein sequence and simulation of protein hydrolysis by the combined action of specific proteases. The BIOPEP database is an open access tool for bioactive peptide research (http://www.uwm.edu.pl/biochemia/index.php/pl/biopep) [49,50,92].

In the past three decades, a number of bioactive peptide sequences associated with differential functional properties and health beneficial activities, have been identified. Bioactive peptides derived from food proteins have the potential to affect the major body systems, namely the cardiovascular-, digestive-, immune-, endocrine- and nervous system and to exert different activities (antihypertensive-, antimicrobial-, antithrombotic-, immunomodulatory-, opioid-like, antioxidant- and mineral binding activity). More recently, new classes of peptides with an impact on energy balance and plasma glucose homeostasis, have been identified. Among dietary proteins, dairy proteins have long been investigated for their health beneficial effects and most likely can be regarded as the most richest source of currently known bioactive peptides [2,3,4,8,24,85,93].

The potential health benefits of dietary protein-derived peptides have long been a subject of commercial interest. The characterization of bioactive peptides released during GI digestion of traditional foods may offer a promising approach for the promotion of health by means of tailored diets. Moreover, bioactive peptides can be incorporated in the form of ingredients in functional and novel foods, dietary supplements and pharmaceuticals with the purpose of delivering specific health benefits [2,3,4,8,85].

Research through in silico analysis, in vitro biochemical assays, cell cultures and in vivo studies via experimental animals, has achieved remarkable results in terms of identification of bioactive peptides from food proteins and exploration of their targets within body systems, their mechanism of action and finally their potential beneficial effects on human health. Research has also been aimed at improving methods for generating bioactive peptides from traditional foods and from protein-rich by-products of food processing. However, despite some clinical studies in humans, the clinical efficacy of most of the currently known bioactive peptide sequences, is still questionable and further investigation is required. Moreover, the administration via oral route in conjunction with a low gastrointestinal bioavailability, may represent a significant limiting factor in the commercial application of related products.

Focusing on the role of food proteins in the regulation of energy homeostasis, a number of dairy protein-derived peptides with the ability to modulate food intake, body weight gain and glucose homeostasis, have been isolated and well-characterized. Again, their real contribution to the metabolic impact of dairy protein intake is in dispute [3,4,5,8,24,85,93]. To this end, it is useful to review some experimental studies, mainly published in the last decade, which support a prominent role of distinct peptidic fragments in conjunction with the role played by the high content of essential amino acids like BCAAs of specific dairy proteins. 

Intact dietary proteins can directly stimulate the enteric hormone release in vitro and may be more potent than the products of their complete hydrolysis [29]. Moreover, distinct proteins may differ in their effects on the release of specific hormones. To this end, whey and pea proteins exerted strongest effects on CCK release whereas codfish, egg and wheat proteins showed more pronounced effects on GLP-1 release from STC-1 cells [29]. β-casein increased secretion of GLP-1 from the same cell line whereas α-casein did not [94]. Finally, intact whey proteins (whey protein concentrate as well as isolated α-lactalbumin or β-lactoglobulin) were shown to significantly increase GLP-1 secretion from STC-1 cells. This secretagogue activity was lost after simulated gastrointestinal digestion [95,96,97]. Actually, it is worthy remarking that some discrepancies may exist between the conclusions from distinct studies, mostly linked to the use of different cell lines or different digestion protocols. In contrast to the effects observed by Rafferty and coworkers [94], in other works, intact α-casein was shown to be more effective at stimulating GLP-1 release than β-casein [98]. Furthermore, the use of a whole simulated gastrointestinal digestion of whey proteins (from enzymatic digestion to colonic fermentation) showed that hydrolyzed samples were more potent CCK- and GLP-1-secretagogues compared to fermented samples and intact proteins in STC-1 cell cultures [99], in contrast with some previously mentioned reports. Anyway, studies performed by using ileal segments of porcine intestine confirmed the stimulatory effect of intact casein on GLP-1 and also peptide YY secretion in vitro [100]. 

Although the in vitro activity of intact proteins on secretory cells of the enteric mucosa may have no physiological relevance because dietary proteins are sorted to degradation upon ingestion, these data support the hypothesis that distinct structural features in a given dietary protein may positively affect its ability of interacting with surface receptors on the enteroendocrine cells and inducing the release of a given hormone. In summary, not only the relative content of specific amino acids to be released after extensive hydrolysis but also the spatial proximity between amino acid residues in the secondary and tertiary structure of a given protein, may be relevant to its interaction with the enteroendocrine elements.

Dietary protein hydrolysates can directly stimulate the enteric hormone secretion tested in vitro and their secretagogue activity is dependent on the protocol used for the enzymatic digestion. More in detail, it has been shown that the GLP-1 secretion by STC-1 cells in response to sodium caseinate, increased gradually over time during simulated gastrointestinal digestion, with the contribution of both gastric- and duodenal phase [8,101]. On the other hand, the simultaneous exposure of STC-1 cells to native casein and trypsin for 30 min further increased the secretion of CCK compared to the effect of native casein alone, without affecting the GLP-1 response [29]. Hence, by varying the hydrolytic conditions (i.e., extensive versus partial hydrolysis), it is possible to convert a dietary protein into different products, i.e., large peptidic fragments versus free amino acids, which will affect the secretion of distinct enteric hormones in a different manner. Some reports suggest that CCK expression and secretion are enhanced by peptides derived from food protein hydrolysis, to a higher extent than by a free amino acid mixture [12,30,102,103].

Although the in vivo gastrointestinal digestion of food proteins cannot be changed, their preliminary exposure to specific proteolytic enzymes from plants or microbes or to microbial fermentation before ingestion, may significantly alter the substrates that will undergo gastrointestinal digestion, thus affecting the pattern of final products in the gut and their potential bioactivity [2,3,4,5,85,93]. This consideration is particularly relevant to milk proteins in a variety of dairy products [104].

STC-1 cells have been used to identify bioactive fractions among casein- or whey protein hydrolysates generated by enzymatic hydrolysis or bacterial fermentation. A GLP-1- and/or CCK-secretagogue activity was found to be associated with peptide-containing fractions, not to the free amino acid component of the hydrolysates. Actually, the individual peptide sequences have not been mostly identified [2,8,85]. 

By using a different cell model, Schellekens and coworkers identified a fraction of sodium caseinate hydrolysate capable of activating the serotonin 5-HT_2C_ receptor subtype [105]. This receptor is known to play a main role in the serotonin pathways modulating food intake. Human embryonic kidney cells (HEK293) expressing 5-HT_2C_ receptor were used for the in vitro assay. The receptor response to the hydrolysate fractions was recorded by measuring calcium influx. 

A chymosin hydrolysate of sodium caseinate with secretagogue activity in vitro, was shown to reduce food intake after intraperitoneal (ip) administration in rodents [106]. However, it did not significantly change food intake, post prandial levels of GLP-1 and insulin in vivo compared to unhydrolysed sodium caseinate after oral administration in both animals and human subjects [8,101], suggesting that the active fractions generated by the preliminary digestion were not resistant to gastrointestinal proteases. 

Preclinical in vitro and in vivo studies are also useful to characterize the activity of specific peptide sequences that are released during the enzymatic digestion of a given protein. To this end, purified-or chemically synthetized peptides can be used. 

In the last decade, our research group contributed to the characterization of whey protein-derived peptides with biological activities related to the control of glycemia or food intake, namely peptides with dipeptidylpeptidase-IV (DPP-IV) inhibitory activity and peptides stimulating the CCK secretion from STC-1 cells [30,49,50]. As to the second class of peptides, we focused our analysis on short peptides (from two to five amino acid long) containing BCAAs, that may be released during GI digestion of β-LG. Indeed, the short-term appetite suppression caused by whey protein intake has been partially attributed to the high content of BCAAs, mainly l-leucine, of β-LG and other proteins [5]. Moreover, β−LG is the most abundant whey protein found in the milk of cow and other ruminants [91,93]. We neglected to compare the effects of the peptides to those of a free BCAA supplement because we treated the cells in a commercial medium (Dulbecco’s modified Eagle medium, DMEM) with an optimized amount of free essential amino acids. 

In our experimental conditions, dipeptides and tripeptides containing only amino acids with aliphatic side-chains (l-alanine, l-proline and the branched-chain amino acids l-leucine, l-Isoleucine, l-valine), were totally ineffective. On the other hand, the pentapeptide ALPMH significantly increased the CCK secretion from STC-1 cells compared to the control (DMEM vehicle). A peptide with a scramble sequence (PHLMA) showed the same effect as ALPMH, suggesting that the length of the peptides and the presence of certain amino acids rather than the specific sequence, were associated with the secretagogue activity on enteroendocrine cells [30]. ALPMH is better known as a peptide with angiotensin-converting enzyme (ACE) inhibitory activity and has been first isolated from β-LG hydrolysates obtained by using pepsin and trypsin [91]. Hence, it may be released upon β-LG GI digestion.

In our experiments, both α-lactalbumin (α-LA) and β-LG hydrolysates prepared in conditions mimicking the gastrointestinal enzymatic digestion, were equally effective at inducing the CCK release from STC-1 cells and were more effective than each peptide tested, suggesting bioactive synergies between multiple components within the hydrolysates. 

In the same study, we ascertained that two more peptides composed by six- and eight amino acids, respectively and not derived from whey proteins, strongly stimulated the CCK release from STC-1 cells in the same concentration range as the pentapeptides ALPMH and PHLMA (millimolar range). The penta-, esa- and octapeptides were also more heterogeneous in their composition compared to shorter peptides, that were all ineffective. They contained both hydrophilic- and hydrophobic amino acids, aliphatic- and aromatic amino acids. 

In conclusion, our data provided further evidence that peptidic fragments derived from partially digested food proteins can have a major impact on CCK release compared with free amino acids or dipeptides. Actually, the length of the peptides and the presence of amino acids with different chemical features within their sequence, may be crucial to their activity on enteroendocrine cells, rather than a specific sequence [30]. 

These data are in agreement with the conclusions of two previous reports showing that intact or partially digested dietary proteins may exert stronger effects on satiety hormone release compared to protein hydrolysates and synthetic peptides [29], and that dipeptides and tripeptides may be ineffective in altering CCK release from STC-1 cells [107]. Furthermore, as to the possible synergistic effects of amino acids with different side chains, it has been previously observed that a mixture of l-tyrosine, l-threonine and l-methionine induced a significant change of CCK release whereas a single amino acid of the mixture had no significant effects [108]. 

Peptide YPFPGPI, so called β-casomorphin-7 (β-CM7), is interesting due to its pattern of biological activities and its resistance to degradation in the GI tract. β-CM7 is basically known as an opiod receptor agonist [8,93]. The interaction with the opioid receptors in the gut mediates its action of delaying gut transit, as observed in animal experimental models [109]. This peptide was also shown to increase the release of somatostatin from endocrine cells in the gut mucosa [110]. Somatostatin, in turn, has a primary role in regulating endocrine and exocrine secretion. In detail, somatostatin contributes to reduce the gastric acid secretion and gastric motility and inhibits the secretion of multiple gastroenteric- and pancreatic hormones [111]. Finally, β-CM7 and its fragment FPGPI stimulated the CCK secretion from STC-1 cells in vitro, whereas smaller fragments (YP and GPI) did not [8,107]. 

β-CM7 and its derivative FPGPI have been identified in the human jejunum after ingestion of a casein preload. FPGPI and other derivatives were also found in yogurt as products of bacterial fermentation [112]. In vitro permeability assays across Caco-2 cell monolayer (an in vitro model of intestinal epithelial barrier) suggested the cleavage of β-CM7 by brush border membrane endopeptidases, and the release of FPGPI into both apical and basolateral medium [107]. Hence, we may conclude that there is evidence of an actual role of this peptide and its derivatives in mediating some effects associated with dairy product ingestion.

In this context, the β-CM7 bioactivity is associated to the claim of differential functional properties between A1- and A2 milk. Most of bovine milk contains two genetic variants of β-casein, the A1- and the A2 β-casein. A2 milk is obtained by animals expressing the A2 variant, only. β-CM7 is released upon digestion of A1 casein, only and is absent in A2 milk and its derivatives. Due to the actions of β-CM7 on digestive functions, A2 milk is claimed to be better tolerated compared with A1/A2 milk in some people. More in detail, in rodents, A1 β-casein relative to A2 β-casein was shown to slow down the transit of food through the digestive system and to induce a pro- inflammatory effect in the colon, which are both opioid-receptor mediated effects. Moreover, A1 β-casein relative to A2 β-casein caused the up-regulation of the enzyme DPP-IV in the small intestine mucosa, a non-opioid effect [53]. In summary, some negative outcomes associated with milk consumption may be attributed to the opioid peptide β-CM7, derived from A1 β-casein. β-CM7 exerts its function by binding to the μ-opioid receptors in the body. It has been suggested that the activation of the μ-opioid receptors in the gut can alter gut microbial composition, impair gut barrier integrity and bile acid metabolism, in addition to increasing gastrointestinal transit time and gut inflammation. In conclusion, BCM-7 might have multiple functions pertinent to human health but the evidence is still limited and further clinical studies are required for confirmation [113]. Moreover, we may add that, taking the effects on the gastrointestinal functions into account, the actual impact of β-CM7 on metabolic health may be different in lean- *vs* overweight or obese subjects.

Glycomacropeptide (GMP) is a large k-casein-derived peptide released by the action of chymosin during cheese-making, and a component of the whey protein fraction. GMP has five potential glycosylation sites. Its carbohydrate-free form is called caseinmacropeptide (CMP). The effects of GMP and CMP on food intake and enteric hormone secretion have been explored in vitro and in vivo in rodents and humans [8,93,114]. GMP was shown to induce the CCK secretion from isolated rat duodenumjejunum [115]. More recently, studies in mice suggested that GMP intake might also have an indirect effect on satiety by altering the gut microbiota and promoting the growth of bifidobacteria and lactobacilli which, in turn, would enhance the secretion of satiety hormones like peptide YY, GLP-1, CCK [116]. Actually, a 7-week long feeding trial with whey protein isolate (WPI) compared to WPI supplemented with GMP, did not show any difference in body weight gain between the two groups of rats. However, in the same experiment, the plasma insulin levels were lowered by the addition of GMP to the WPI, suggesting a beneficial effect on insulin sensitivity [117]. As to human subjects, the results of clinical trials have been contradictory [8]. In summary, GMP was shown to contribute to the appetite-suppressant effects caused by whey protein intake in healthy humans [118,119] but GMP intake did not significantly change GLP-1 levels, CCK levels, food intake, and subjective appetite rating compared to GMP-depleted whey proteins in overweight and obese men [120,121]. Moreover, a CMP-containing beverage did not significantly affect *ad libitum* food intake in healthy subjects and GMP intake did not alter inflammatory parameters, gut microbiota and body weight in healthy adults [122]. In summary, preclinical studies and some clinical trials suggest that GMP can be effective after oral administration and the carbohydrate chains may play an important role in its interaction with enteroendocrine cells. Actually, a primary role of this specific component in mediating the effects of whey protein intake on food intake regulation, glucose homeostasis and microbiota composition [66,123], is still controversial.

Dipeptide RF is found encrypted in several peptide sequences released by the action of chymosin on sodium caseinate [106]. RF is able to induce the CCK release from STC-1 cells in vitro and revealed to be effective also in vivo in mice. Intraperitoneal (ip) administration of RF reduced cumulative food intake in the two hours post injection. When administered by oral gavage, it delayed the gut transit of food [8,124]. 

In the last decade, our research group and others have investigated the possible release of bioactive peptides inhibiting the aminopeptidase dipeptidylpeptidase-IV (DPP-IV) activity, upon enzymatic hydrolysis of dairy proteins and other food proteins [49,50,51,52,85,125]. 

DPP-IV is responsible of the incretin (GLP-1 and GIP) rapid inactivation after meal. Thus, DPP-IV inhibitory compounds can enhance the incretin activity and ameliorate the control of plasma glucose levels. Indeed, synthetic compounds acting as DPP-IV inhibitors (so called gliptins) reduced the clearance of GLP-1 and GIP and are currently a therapeutic option for the treatment of type 2 diabetes. These drugs are orally administered and exert their action upon absorption into systemic circulation [43,46,47,48]. 

A large number of peptides encrypted in caseins and whey proteins and ranging in size from two to about fourteen amino acids, have been characterized as DPP-IV inhibitors in vitro. Among this class of bioactive peptides, some sequences have been predicted to be released upon digestion of a specific dairy protein in the gut, according with the results of an in silico simulated hydrolysis with GI proteases. Some peptides have been isolated from hydrolysates prepared by using proteases in vitro [49,50,51,52,85,125,126]. 

Although some structural features may confer resistance to proteases (i.e., the tripeptides IPP and IRW are naturally resistant to digestive proteases) [127], the bioavailability of orally administered peptides and peptides derived from food protein digestion, is expected to be scarce due to degradation in the GI tract and limited absorption across the intestinal epithelial barrier. Hence, the efficacy of DPP-IV inhibitor peptides in vivo has been in dispute [3]. However, some considerations may reinforce an interest in this class of bioactive peptides. First, evidence has been provided of the stability and/or bioactivity of a few specific DPP-IV inhibitory peptides in vivo. Second, DPP-IV is expressed as a cell-surface protein by a number of cell types including endothelial cells, immune cells and the brush border of the intestinal mucosa. It is also expressed by some components of the microbiota in the gut lumen. Moreover, incretins are expected to be largely inactivated by DPP-IV in the basolateral medium of the enteroendocrine cells in the intestinal mucosa, before being absorbed and entering into the systemic circulation [43,45,46,47,48]. We might want to conclude that food-derived DPP-IV inhibitors may act locally as endogenous inhibitors in the gut reducing DPP-IV activity at different levels (i.e., in the proximal small intestine) [128] with consequences that are still to be explored. 

As to the experimental data suggesting that some milk protein-derived peptides with DPP-IV inhibitory activity in vitro, may be also active in vivo, we can say that a few peptides among those tested (LKPTPEGDL, LPYPY, IPIQY, IPI, WR) were shown to cross Caco-2 cell monolayers in vitro [126,129]. Moreover, three β-LG-derived inhibitors (IPAVFKIDA, IQKVAGTW, LKPTPEGDLE) were identified in the stomach of infants 2 h post administration of mother’s milk supplemented with infant formula [8,130]. The *N*-terminal fragment of the first peptide (IPAVF) had been previously isolated from a trypsin hydrolysate of β-LG and characterized as a DPP-IV inhibitor with a low IC50 value in vitro [131]. Hence, based on the two reports, there is a chance that IPAVF is a product of the gastrointestinal digestion of β-LG. Two more peptides with DPP-IV inhibitory activity (PPVPQ and IPM) were identified in human GI tract after milk consumption [52]. A β-casein peptide with DPP-IV inhibitory activity (LPQNIPPL, IC_50_: 46 µM) was isolated from a water-soluble extract of Gouda cheese. Its administration reduced the postprandial plasma glucose levels in female rats during a glucose tolerance test [8,132]. Finally, among the most potent DPP-IV inhibitors isolated from milk protein hydrolysates, it is worth mentioning some tryptophan-containing dipeptides (WR, WK, WL) [133]. Taking their structure into account, their bioavailability after milk consumption should be further investigated because dipeptides and tripeptides may be expected to cross the intestinal mucosa and reach the systemic circulation in an intact form [126]. At present, we can say that the dipeptide WR was shown to cross an intestinal epithelial cell monolayer in vitro [126,129].

Recently, novel evidence supporting the hypothesis that dietary peptides can inhibit DPP-IV activity in vivo to reduce the degradation of GLP-1 and potentiate the GLP-1 response to food intake, has been reported by Shimizu and coworkers [134]. The authors compared the GLP-1 response to the oral intake of a carbohydrate versus a lipid and versus two different proteins (casein fraction and whey fraction) in normal rats, in DPP-IV-deficient rats and normal rats treated with the DPP-IV inhibitor sitagliptin. All these stimuli should induce the GLP-1 secretion in rats. Actually, the absence of DPP-IV activity or its partial pharmacological inhibition improved the GLP-1 response to the carbohydrate-, the lipid- and the casein intake whereas the increase of GLP-1 levels induced by whey proteins was not significantly changed. The authors concluded that the intake of whey proteins was *per se* related to a decrease of DPP-IV activity in rats and a consequent decrease of GLP-1 degradation [134].

α-glucosidase is a possible target of compounds released upon food digestion in the small intestine and acting locally at the level of the brush border of the enterocytes, before being absorbed or entirely degraded [85].

α-glucosidase cleaves glycosidic bonds in complex carbohydrates to release absorbable monosaccharides. From a pharmacological point of view, the α-glucosidase inhibition represents a strategy to decrease the postprandial hyperglycemia in type 2 diabetes by retarding the absorption of glucose. It has been suggested that the α-glucosidase inhibition may also have an impact on GLP-1 secretion after meal. Indeed, the enzyme inhibition delays the monosaccharide absorption, with the outcome of an increase in free sugar levels in the lower intestine, which in turn will enhance the release of GLP-1 from l cells. Acarbose and miglitol are well-known α-glucosidase inhibitors [85].

Various foods have been reported to be a source of compounds with α-glucosidase inhibitory activity, including non-saccharide inhibitors. In this context, some bioactive peptides acting as α-glucosidase inhibitors have been isolated from hydrolysates of sardine muscle and egg white protein. Peptides with the highest affinity to α-glucosidase (RVPSLM, TPSPR) display IC_50_ values in the micromolar range [135]. Also β-LG and whey protein isolate were shown to release α-glucosidase inhibitors during enzymatic hydrolysis in vitro [85,136,137]. The impact of a proprietary milk protein hydrolysate with α-glucosidase inhibiting properties claimed to be linked to the whey fraction and, in detail, to the dipeptide AP, was determined in a trial in prediabetic subjects. The incremental area under the curve of glucose after a meal rich in carbohydrates was significantly reduced by the pre-load compared to placebo with a minor insulinotropic effect, in agreement with a primary action on glucose absorption [86].

Finally, it is worthy to mention a drawback of a prolonged inhibition of α-glucosidase, which is linked to increased carbohydrate fermentation in the intestine and possible effects on the microbiota composition, with consequent flatulence, abdominal cramping and diarrhea.

Three more bioactive peptides derived from β-LG hydrolysates, revealed to be active in vivo in rodents. Two of them decreased food intake in fasted animals after oral administration. The third one increased insulin sensitivity in mice. Their mechanism of action has been partially characterized but it may deserve further investigation.

The nonapeptide LIVTQTMKG has been isolated from the products of enzymatic digestion by thermolysin. It decreased the acylated ghrelin release from a mouse ghrelinoma cell line in vitro, and the plasma ghrelin levels measured one hour post oral administration in fasted mice. The peptide was not effective in non-fasted mice. Actually, this result might be expected because the basal levels of circulating ghrelin are well-known to be markedly lower in non-fasted animals compared to fasted animals. The suppression of ghrelin release in fasted animals may account for the decrease of food intake over four hours post peptide administration [8,138]. However, the peptide was active on food intake in vivo at a dosage which can be regarded as low (1 mg/kg), whereas it decreased the ghrelin secretion in vitro dose-dependently in the micromolar range (10–100 µM), which is high for a peptidic compound and suggests low affinity interactions with the cell system.

Peptide HIRL has been called β-lactotensin and was isolated from the products of β-LG digestion with chymotrypsin. It was characterized as a neurotensin receptor agonist and caused ileum contraction in an ex-vivo system. Intracerebroventricular-, intraperitoneal- and oral administration of this peptide caused a reduction of food intake in fasted mice. The dose required for the oral route (500 mg/kg) was 5-fold higher compared to the intraperitoneal route. The investigation of the molecular mechanism underlying its anorectic effect by the use of selective receptor antagonists, revealed that HIRL may suppress food intake by activating the corticotropin releasing factor (CRF) system in the central nervous system, independently of the activation of the neurotensin receptors NT_1_ and NT_2_ [8,139].

Wheylin-1 is an anxiolytic-like dipeptide (MH) isolated from thermolysin digest of β-LG. This peptide was found to increase insulin sensitivity in vivo in mice and Akt phosphorylation in hepatocytes and skeletal muscle cells in vivo and in vitro. The Akt phosphorylation has a primary role in the intracellular signalling pathway mediating the metabolic effects of insulin, downstream of insulin receptor. In summary, wheylin-1 is a bioactive peptide which may contribute to the positive impact of whey protein intake on glucose homeostasis [140].

Milk protein can also be a source of cyclic dipeptides with a 2,5-diketopiperazine (DKP) structure. These cyclic compounds can be formed by the *N*-terminal amino acid residues of a linear peptide and have been isolated from various foods, with the highest levels found in processed food like roasted coffee, roasted malt, essence, and fermented beverages like beer. They have received considerable attention as bioactive compounds. A DKP compound, cyclo(-His- Pro), displayed some effects in vivo, which include a decrease of food intake and body weight gain in rats [141,142].

Finally, an α-S1-casein-derived tripeptide (YLG) was found to improve cognitive decline in mice fed a high-fat diet. This peptide is released upon digestion of α-S1 casein by gastrointestinal proteases and was effective in mice when orally administered, suggesting a resistance to degradation in the GI tract. TLG administration reversed the decrease in hippocampal neurigenesis and in neurotrophic factor levels caused by the high-fat diet intake for 1 week. It is not known whether the effect of the peptide on neuronal cells was either direct, suggesting not only a good bioavailability but also its transit across the blood-brain-barrier, or indirect mediated by peripheral actions which may include actions on metabolic health [143].

As to the ability of dairy proteins to modulate metabolism and appetite, while most of the experimental investigations have been addressed to identify bioactive derivatives associated with their appetite-suppressant and body weight-decreasing effects and their possible benefits in terms of glucose homeostasis, some studies provided evidence of products of hydrolysis with a reversal function. In this context, a ghrelinergic peptide mixture was found in casein hydrolysates, but specific peptides were not isolated and identified. This mixture was shown to activate the growth-hormone-secretagogue receptor-1a (GHSR-1a), a ghrelin receptor, in a calcium mobilization assay in in vitro cultured cells. In addition, both additive and synergistic effects were observed following the co-exposure of GHSR-1a to both the hydrolysate and ghrelin. GHSR-1a has a role in mediating the stimulatory effect of ghrelin on food intake. Indeed, the administration of the peptide mixture by oral gavage to rats, determined a short-term increase in food intake. On the other hand, the intraperitoneal route was not effective, suggesting a local action in the gut after oral administration. To this end, the authors remarked the expression of GHSR-1a on vagal nerve terminals throughout the small and large intestine. In summary, although further studies are required to better characterize a peptide-mediated ghrelinergic bioactivity in casein hydrolysates, we may remark that the two main milk protein fractions, caseins and whey proteins, can release factors upon digestion which show quite opposite effects on the ghrelinergic system and hence on mechanisms regulating hunger [144].

The activities of the specific peptide sequences mentioned in this section have been summarized in Table 1. 

## 3. Final Considerations and Perspectives 

A low bioavailability of any peptide-based formulation post oral administration, due to rapid degradation by proteolytic enzymes in the GI tract and scarce absorption of protease-resistant peptides across the intestinal epithelial barrier, is a significant limiting factor in the commercial application of bioactive peptide-related products, which include traditional foods or novel foods as a source of bioactive compounds, functional foods endowed with specific health claim beyond their nutritional values and diet supplements with specific formulations.

The research reports reviewed in this article provided evidence that, among a number of food protein-derived bioactive peptides isolated and characterized in in vitro studies, some peptides can be active in vivo post oral administration, suggesting either a local activity in the gut at the level of the brush border of the epithelial barrier and at the level of the gut microbiota or, alternatively, a systemic action on specific molecular targets after entering systemic circulation. 

The mechanisms mediating peptide absorption across the intestinal mucosa barrier include paracellular transport through one or more tight junctions proteins; direct penetration of cell plasma membrane, which is an intrinsic property of some peptides; selective transport by specific transporters; endocytosis or transcytosis by enterocytes [3,26].

Two decades ago, the ACE-inhibitory tripeptides IPP and VPP were found in the abdominal aorta of rats after oral administration of sour milk [4]. VPP was shown to cross a cell monolayer by paracellular and transcellular routes. Now, we can say that also some peptides which are able to interact with the endocrine control of food intake and glucose homeostasis can be absorbed as intact or partially degraded into blood circulation. To this end, the amino acid composition (i.e., the presence of proline within their sequence), the length of the peptide and possible backbone modifications may contribute to confer resistance to degradation by gut proteases. 

In summary, evidence has been accumulated of a role of bioactive peptides in mediating the effects of some food proteins, like whey proteins and other dairy proteins, on metabolic health. Nevertheless, this conclusion does not rule out that some effects may be related to the amino acid composition of these proteins and to the consequent rise of specific amino acids in plasma upon their complete digestion. 

Focusing on the possible role of whey protein supplementation in body weight reduction programs or to improve the postprandial glucose control, it is important to remark that a few clinical trials have suggested the relevance of timing as an additional factor so as to produce the most effective response to whey protein administration before meals or with meals, in terms of health benefits [84,145,146]. We may say that this consideration is consistent with the intrinsic properties of whey proteins, that have been associated with the metabolic response: in detail, the peculiar rate of GI digestion and amino acid absorption (whey proteins are so-called “fast” proteins); consequently, the rapid and temporary rise of BCAAs and other essential amino acids in plasma; the release of bioactive peptides affecting the enteric hormone system, especially the CCK secretion and the incretin system, upon their GI digestion.

It is worth remarking that most dairy protein-derived bioactive peptides associated with potential health benefits have been isolated and characterized starting from native protein mixtures or isolated proteins after simulated GI digestion or microbial fermentation. Actually, we cannot rule out that the processing technologies applied to food (i.e., heating techniques or preparation of emulsions, solid- and liquid foods with the same protein composition) may vary the release of bioactive compounds from a given protein compared to what is expected based on its native structure, by altering the digestion rate and the protein susceptibility to proteases or inducing reactions which involve the amino acid side-chains. Investigating the influence of these technologies on the products derived from the digestion of a dietary protein in the gut, in conjunction with the current knowledge on the bioactive peptide sequences associated with a given functional target, may help to predict the impact of a traditional or a novel food containing that protein on the wanted target (i.e., food intake or plasma glucose control). In regards to this topic, the reader may also refer to a previous review for a more in-depth discussion [12]. We may add that the topic is quite relevant to the use of whey proteins as ingredients of functional foods or supplements to be tested in clinical trials aimed at evaluating their effects on appetite, energy intake, body weight and glucose homeostasis as well as to the possible role of traditional foods containing whey proteins in a dietetic regimen.

Finally, with regard to potential bioactive factors in milk, we have to mention exosomes (Figure 1). Exosomes are a subpopulation of so called extracellular vesicles. This term denotes submicron-sized lipid envelopes that are produced and released from a parent cell and can be taken up by a recipient cell. EVs are capable of mediating cellular signalling by carrying nucleic acids, proteins, lipids and cellular metabolites between cells and organs. There has been increasing interest in their possible role in the development of metabolic diseases [147]. As to the extracellular vesicles called exosomes, the inward budding of endosomal membranes generates intraluminal vesicles in cytosolic multivesicular bodies (MVBs) and the fusion of these MVBs with the plasma membrane releases small vesicles. Exosomes contain macromolecules like peptides, mRNA and microRNAs and can interact with human cells. Exosomes have been isolated from bovine milk and also from whey protein hydrolysates. Recently, it has been provided evidence that the exosomes isolated from whey protein hydrolysates can enhance the protein synthesis in skeletal muscle cells in vitro. The authors concluded that exosomes may have a role in mediating the anabolic effect of whey protein intake on skeletal muscle and suggested an action independent of the mTOR signalling pathway activation and most likely dependent on microRNAs and changes in gene expression [148].

Dietary exosomes may be considered a novel factor *per se* in the functional interaction between foods and biological systems. To this end, bovine milk exosomes significantly alter the gut microbiome in non-bovine species, and bovine milk extracellular vesicles may alter amino acid metabolism in mice [147,149]. Furthermore, although there is no evidence yet of food-derived exosome absorption into circulation in vivo, it would be interesting to investigate a possible role of exosomes as a delivery system protecting bioactive peptides from degradation and facilitating their passage across the intestinal barrier and their delivery to tissue targets.

The isolation and characterization of bioactive peptides derived from food protein hydrolysis in vitro is an exciting research area which offers novel factors for dietary supplements with specific formulations and health claims. Actually, the success in the development of orally administered supplements depends on the availability of delivery systems protecting peptides during gastric transit up to the small or large intestine. Current and future chemical and biotechnological strategies are beyond the aim of this article and have been reviewed elsewhere [8]. Finally, the GI digestion and the integrity of the intestinal epithelial barrier contribute to protect us from undigested or partially digested exogenous compounds. Making a bioactive peptide resistant to hydrolytic enzymes and forcing its transit across the barrier requires excluding potentially damaging effects such as toxicity, allergen activity, and mutagenicity [2], as well as to carefully explore any effect on cell signalling pathways regulating cell growth, differentiation and functionality [30,60,150].

## Figures and Tables

**Table 1:** A summary of bioactive peptides released during the enzymatic digestion of dairy proteins (bovine), and their in vitro and in vivo activities related to the control of food intake and glucose homeostasis.

Source	Peptides	Actions	References
β-lactoglobulin	ALPMH	-stimulates CCK secretion in vitro	[30]
β-casein (A1 genetic variant)	YPFPGPI YPFPGPI FPGPI	-opiod receptor agonist, -delays gut transit, -stimulates somatostatin release in vivo, -other μ-opiod receptor mediated effects in the gut, -up-regulation of DPP-IV espression in the small intestine (non opiod effect), -stimulate CCK secretion in vitro	[109,110] [113] [53] [8,107]
k-casein Whey protein fraction	Glycomacropeptide (GMP)	-stimulates CCK secretion from isolated rat intestine, -stimulates the release of satiety enteric hormones in mice, by altering the gut microbiota composition, -contradictory results as to the effects on appetite, food intake and enteric hormone release in humans,	[115] [116] [118,119,120,121,122]
Caseins Whey proteins	DPP-IV inhibitory peptides LKPTPEGDL, LPYPY, IPIQY, IPI, WR LPQNIPPL	-inhibit DPP-IV protease activity in vitro -possible delay of incretin hormone (GLP-1 and GIP) degradation and improvement of the control of postprandial glucose levels (systemic effect), -possible local action as inhibitors of DPP-IV activity in the gut (brush border of intestinal mucosa and gut microbiota) No clear evidence of their passage across the intestinal epithelial barrier into circulation -DPP-IV inhibitors in vitro, can cross an epithelial cell monolayer (Caco-2 cells) in vitro -DPP-IV inhibitor in vitro, improves postprandial glucose control in rats	[49,50,51,52,125,126] [134] [126,129] [8,132]
β-lactoglobulin Whey protein isolate	α-glucosidase inhibitory peptides AP and other peptides (not isolated and identified)	-inhibit α-glucosidase activity in vitro -inhibit α-glucosidase activity in vitro, -delay glucose absorption after meal in prediabetic subjects	[85,136,137] [86]
β-lactoglobulin	LIVTQTMKG	-decreases acylated ghrelin release in vitro, -decreases plasma ghrelin levels and food intake in fasted mice after oral administration	[138]
β-lactoglobulin	HIRL	-reduces food intake in fasted mice after oral-, ic- or ip administration	[139]
β-lactoglobulin	MH	-anxiolytic-like dipeptide, -increases insulin sensitivity in mice and Akt phosphorylation (insulin receptor signalling) in hepatocytes and skeletal muscle cells in vitro and in vivo	[140]
α-S1-casein	YLG	-reverses cognitive decline in mice fed a high-fat diet after oral administration (possibly direct effects on neuronal cells or indirect effects mediated by peripheral actions, including actions on metabolic health)	[143]
caseins	Ghrelinergic peptide mixtures	-activate ghrelin receptor (GHSR1a) in vitro -induce a short-term increase of food intake in rats after oral administration (not so after ip administration)	[144]
sodium caseinate	RF	-stimulates CCK secretion in vitro, -delays gut transit of food after oral administration and decreases food intake after ip administration in mice	[8,106,124]

Abbreviations: ip, intraperitoneal; ic, intracerebroventricular; GLP-1, glucagon-like peptide-1; GIP, glucose-dependent insulinotropic peptide; CCK, cholecystokinin.

## Abbreviations

α-LA	α-lactalbumin
β-CM7	β-casomorphin-7
β-LG	β-lactoglobulin
CCK	cholecystokinin
CMP	caseinomacropeptide
DPP-IV	dipeptidyl peptidase-IV
GCPR	G-coupled protein receptors
GI	gastrointestinal
GIP	glucose-dependent insulinotropic peptide
GMP	glycomacropeptide
GLP-1	glucagon-like peptide-1
ip	intraperitoneal
os	oral
WPC	whey proteinconcentrate
WPI	whey protein isolate
